# Sleep disturbances after an ultra-trail endurance race: a field study

**DOI:** 10.1016/j.sleepx.2026.100197

**Published:** 2026-07-28

**Authors:** Fanny Fachan, Franck Vilcourt, Bruno Lemarchand, Florian Chouchou

**Affiliations:** a IRISSE Laboratory, EA4075, Faculty of Human and Environmental Sciences, University of La Réunion, Le Tampon, France; b Sports Medicine Unit, University Hospital of La Réunion (CHU), Saint-Pierre Hospital Site, France; c Jean Monnet University, NEUROPAIN Team, CRNL, Inserm CNRS, CHU Hôpital Nord, Saint-Etienne, France

**Keywords:** Sleep, Running, Ultra-trail, Endurance, Actigraphy

## Abstract

**Background:**

Endurance running has a substantial influence on sleep, while insufficient or disturbed sleep prior to exercise may impair endurance performance. However, these interactions remain poorly characterized under real-life conditions. The aim of this field study was to examine sleep patterns in athletes before and after an ultra-trail race.

**Methods:**

Eighteen athletes (mean age 34.6 ± 8.13, 3 women) underwent an ultra-trail race at 03:30 a.m. (Ultra Marathon des Cirques, La Réunion, France, 64 km, 5000 m of elevation gain, June 2024). After completing various questionnaires to assess their sleep, all participants filled out a sleep diary and all wore an actimeter for six nights before and after the event.

**Results:**

The athletes reported modest subjective sleep alterations, mainly a later wake-up time and reduced sleep quality on the night following the race (p < 0.05). However, actigraphy-based sleep measurements revealed objective sleep changes, including reduced sleep efficiency (p < 0.05), longer nocturnal wake time (p < 0.05), an increased number of awakenings (p < 0.05), and wake after sleep onset (p < 0.05), lasting up to four nights after the race.

**Conclusions:**

Participants reported minor sleep complaints following the ultra-trail, whereas more pronounced sleep disruptions can persist for up to four nights postrace. These disruptions are characterized by sleep fragmentation and reduced sleep efficiency. The mechanisms underlying these alterations remain to be elucidated and may involve factors such as sleep deprivation, cumulative fatigue, hypoxia, and hyperthermia.

## Highlights

• The predominant changes observed in athletes' sleep are a decrease in sleep efficiency accompanied by an increase in sleep fragmentation.
• Even though objective modifications occurred, only minimal subjective changes were observed postrace in endurance athletes.
• Objective sleep disruptions persist last up 5 nights postrace.

## Graphical abstract

**Figure:**
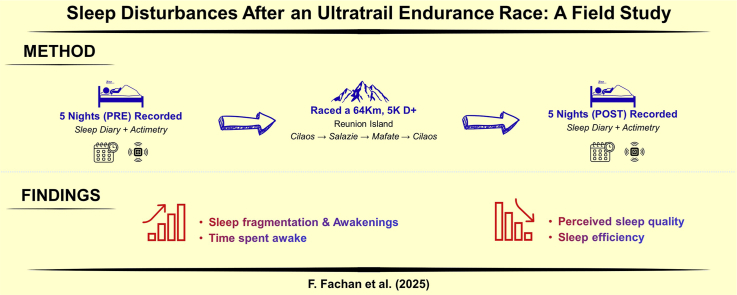

## 1 Introduction

Sleep and exercise exert reciprocal effects mediated by intricate, bidirectional interactions [1,2]. Experimental studies conducted in laboratory settings have demonstrated that acute sleep deprivation, whether total [3] or partial [4], has a detrimental effect on aerobic exercise performance [5]. This deleterious effect appears to occur notably through an alteration in the perception of effort, particularly by increasing perceived exertion [6,7]. Conversely, promoting sleep, especially over multiple days, has been shown to enhance endurance performance in improving perceived exertion and attention [8,9].

Aerobic physical exercise is well known to exert a substantial influence on sleep [1,10]. Acute modifications are typically observed, including increases in total sleep duration [[11], [12], [13], [14], [15], [16]] and in the proportion of deep sleep stages, such as slow-wave (SW) [[11], [12], [13], [14], [15], [16]] and a decrease of paradoxical (rapid-eye-movement (REM)) sleep [[11], [12], [13],^15^,^16^]. However, findings across studies remain heterogeneous, likely due to exercise-related factors such as the duration [11,12,[14], [15], [16]] and intensity [[11], [12], [13], [14], [15]]. Individual characteristics, including age [11,12,14] and athletic level [11,12,[14], [15], [16]] may also modulate these effects.

Considering outdoor activities, the number of trail and ultra-race races has grown rapidly over the past decade [17], offering a wide range of distances and elevation profiles from short trail races ranging from 21 to 41 km to ultra-trail races over 42 km. These outdoor events also often extend over several days, adding the additional challenge of sleep deprivation to the inherent demands of endurance running and environmental constraints, including hypoxia and exposure to heat and/or cold [18,19]. All these constraints inevitably lead to changes in sleep following this type of event. In an early study on a small sample, Shapiro et al. (1981) observed major changes in sleep SW and REM sleep durations after an ultra-trail road race in six highly fit athletes on four successive polysomnographic nights after completing a 92-km road race [20]. Interestingly, these recordings reveal an increased wake duration after the trial. However, the origin of this wakefulness remains unidentified. One way to understand the sleep changes that follow an ultra-trail event is by considering the homeostatic dimension of sleep [21], namely the increase in sleep pressure that can be caused by sleep deprivation [22,23], heat exposure [24] and hypoxia [25], and the physical demands of endurance exercise [20]. More modestly, the circadian process may also contribute depending on the race start time and its duration [26]. However, the homeostatic or circadian processes alone may not fully account for post-race sleep alterations. A long wake after sleep onset (WASO) raises the question of arousal process [[27], [28], [29]] due to ultra-endurance exercise. All these constraints and the stress they generate can also disrupt sleep continuity, promoting fragmented and less restorative sleep than would be necessary [30]. An integrative framework therefore could consider the interaction between homeostatic sleep pressure, circadian processes regulating the timing of sleep and wakefulness [21], and arousal-related process, which encompass exercise-induced physiological stressors that can destabilize sleep [31,32]. These processes may act in opposing directions and could help explain why an increased need for sleep after ultra-endurance events does not necessarily translate into long and continuous sleep.

Given the limited number of field studies addressing these questions, it seemed necessary to explore this contradiction among athletes participating in this type of trail-running event using equipment that minimally disrupts sleep. Sleep changes can manifest as both subjective alterations in perceived sleep quality [33] and objective sleep measurements including the sleep duration, the number of awakenings, WASO and measures of sleep efficiency measured by actimeter [34]. Combining these subjective and objective approaches enables a more comprehensive understanding of sleep alterations in response to ultra-endurance races [33]. Accordingly, the aim of this study was to investigate sleep through objective and subjective sleep parameters before and after an ultra-trail race. We hypothesized that, despite the increased need for sleep, the extension of sleep duration after an ultra-trail event coincides with sleep disruptions, including sleep fragmentation and efficiency.

## 2 Methods

### 2.1 Participants

Eighteen participants (mean age 34.6 ± 8.13, 3 women) were recruited for this study. All participated in the Ultra Marathon des Cirques (UMC) race (64 km, 5000 m of elevation gain, on June 26, 2024, in Cilaos, La Réunion, France at 03:30 a.m.). The inclusion criteria for the study were as follows: participants had to be over 18 years old, registered for the UMC solo race, regular practitioners of trail and ultra-trail running, fluent in French, and residents of La Réunion. Exclusion criteria included no current use of sleep medication, no diagnosed sleep disorders, no engagement in night shift work, no history of mental or psychiatric conditions, and no known allergy to electrode gel or electrodes. The recruitment process began six weeks before the race with an e-mail invitation sent to all 934 registered participants, asking them to complete a general questionnaire covering demographic information and training habits. Among the 221 respondents (a 24 % response rate), 56 met the sleep-related eligibility criteria. After a thorough assessment of all inclusion and exclusion criteria, 20 participants were ultimately deemed eligible for the study (n = 20). One month prior to the race, participants provided informed consent and underwent a thorough evaluation of the inclusion and exclusion criteria. The study was approved by the Ethics Committee (24-A01243-44) and performed under informed consent according to the Declaration of Helsinki.

### 2.2 Study design

The study covered 6 nights (from N-5) before the event (including the night of the race start, N0) and 5 nights (N+5) after the UMC race. Participants wore actimeters and completed a sleep diary twice daily (morning and evening) during the six nights before and the five nights after the race [35,36]. Participants completed a series of questionnaires to study their sensation of fatigue, their sleep, their anxiety and depression [35,[37], [38], [39]].

### 2.3 Data recordings and analysis

#### 2.3.1 Actimetry

Wearable sensors (e-Tact, BodyCap Medical, France) continuously recorded participants' sleep–wake cycles throughout the study [40,41]. Participants were instructed to continuously wear the device on their nondominant arm throughout the study period, removing it solely for showers and replacing the adhesive film daily. They were advised to avoid removing the device except on race day or in the event of skin irritation, in which case they were to notify the experimenter.

The device was configured to record activity data with sampling frequency at 50 Hz, with a 1-min measurement period, a sensitivity threshold of 0.1 g, and a recording range of 2 G using the time above threshold (TAT) method [40]. Sleep/wake phases were analysed daily for a total of 11 d (6 d before and 5 d after the race). The analysis parameters were set as follows: high activity above 500 TAT, moderate activity between 250 and 500 TAT, and low activity below 250 TAT, with high sensitivity to movements during sleep phases. Several parameters were extracted to quantify objective sleep changes including total sleep duration, and sleep disruption, reflected by sleep efficiency, number of awakenings, proportion of the sleep period spent awake, and WASO.

#### 2.3.2 Sleep diary

The sleep diary was used as a simple and effective tool for reporting bedtime, wake-up time, naps, and a subjective assessment of sleep quality, wake-up and daytime alertness giving an overview of sleep organisation [42] and subjective sleep alterations [43]. When used over several weeks, the sleep diary provides detailed insight into a subject's sleep organisation and pattern. This tool is notable for its simplicity, low cost, and informative value providing a reliable estimate of bedtime and wake-up time [43], making it a frequently used instrument in the screening of primary insomnia [44]. Instructions were provided when the actimeter was distributed two days before the study began. Participants were asked to fill it out systematically every morning and evening to minimise recall bias.

#### 2.3.3 Questionnaires

Participants completed a series of questionnaires including the French version of the Pittsburgh Sleep Quality Index assessing sleep over the past month, comprising of 24 questions, with a score above 5 indicating poor sleep quality [45]; the Insomnia Severity Index evaluating the presence and severity of insomnia where a score above 15 indicates clinical insomnia [46]; the Fatigue Severity Scale using 9 items to measure fatigue severity and its daily impact, where a higher score reflects more severe symptoms [47]; and the Hospital Anxiety and Depression Scale, where a score above 11 indicates certain symptoms [48]. Additionally, two questionnaires were used to gather demographic data and training habits.

### 2.4 Statistical analysis

Statistical analyses were performed using SPSS software version 25 (IBM, Chicago, IL). The normality of data distribution was assessed using skewness and kurtosis [49]. If necessary, a logarithmic transformation was applied to normalize the data. Subsequently, two-sided repeated measures analysis of variance (ANOVA) was conducted to examine the within-subject time effects (from night-to-night changes). Greenhouse-Geisser correction of degrees of freedom was applied and Tukey post hoc tests were performed when appropriate between the average of the nights before the race considered as an overall baseline and each individual night before and after the race. Statistical significance was defined as p < 0.05.

## 3 Results

Of the 20 participants enrolled, 18 (mean age 34.6 ± 8.1 y, 15 men – 83.3 %, 3 women – 16.7 %) were included in the analysis. Two participants were excluded from the analysis, one for withdrawing consent and the other for an injury incurred during the race. The participants completed the 64 km and 5000 m elevation gain course in an average time of 13.8 ± 5.8 h. All volunteers reported no sleep problem with habitual sleep duration between 6 and 8 h (72.2 %), and no problem related to fatigue, anxiety and depression. The majority of participants had been practicing trail running for one to three years (61.1 %) and had participated at least once in races ranging from 30 to 100 km. Demographic characteristics are summarized in Table 1.

**Table 1:** **Demographic characteristics of the participants.** Data are presented as mean ± standard deviation or as number (percentage). BMI: Body Mass Index; PSQI: Pittsburgh Sleep Quality Index; ISI: Insomnia Severity Index; FSS: Fatigue Severity Scale; HAD: Hospital Anxiety and Depression Scale. Distance indicates the proportion of participants who had previously completed races of each distance.

Variables		All volunteers (n = 18)
Sex (men, %)		15 (83.3)
Age (years)		34.6 ± 8.1
BMI (kg/m^2^)		21.5 ± 2.0
PSQI (/21)		5.6 ± 2.3
ISI (/28)		5.6 ± 6.2
FSS (/9)		2.4 ± 1.4
HAD – Anxiety (/21)		1.4 ± 1.6
HAD – Depression (/21)		1.5 ± 2.3
Total race time (hours)		13.8 ± 5.8
Reported sleep duration in hours (n, %)	5 – 6	3 (16.7)
	6 – 7	5 (27.8)
	7 – 8	8 (44.4)
	8 – 9	2 (11.1)
Years of trail running experience (n, %)	<1	3 (16.7)
	1 – 3	11 (61.1)
	5 – 10	3 (16.7)
	>10	1 (5.6)
Distance (kilometers) of trail running race completed (n, %)	0 – 30	17 (94.4)
	30 – 50	15 (83.3)
	50 – 100	15 (83.3)
	>100	10 (55.6)

**Fig. 1:**
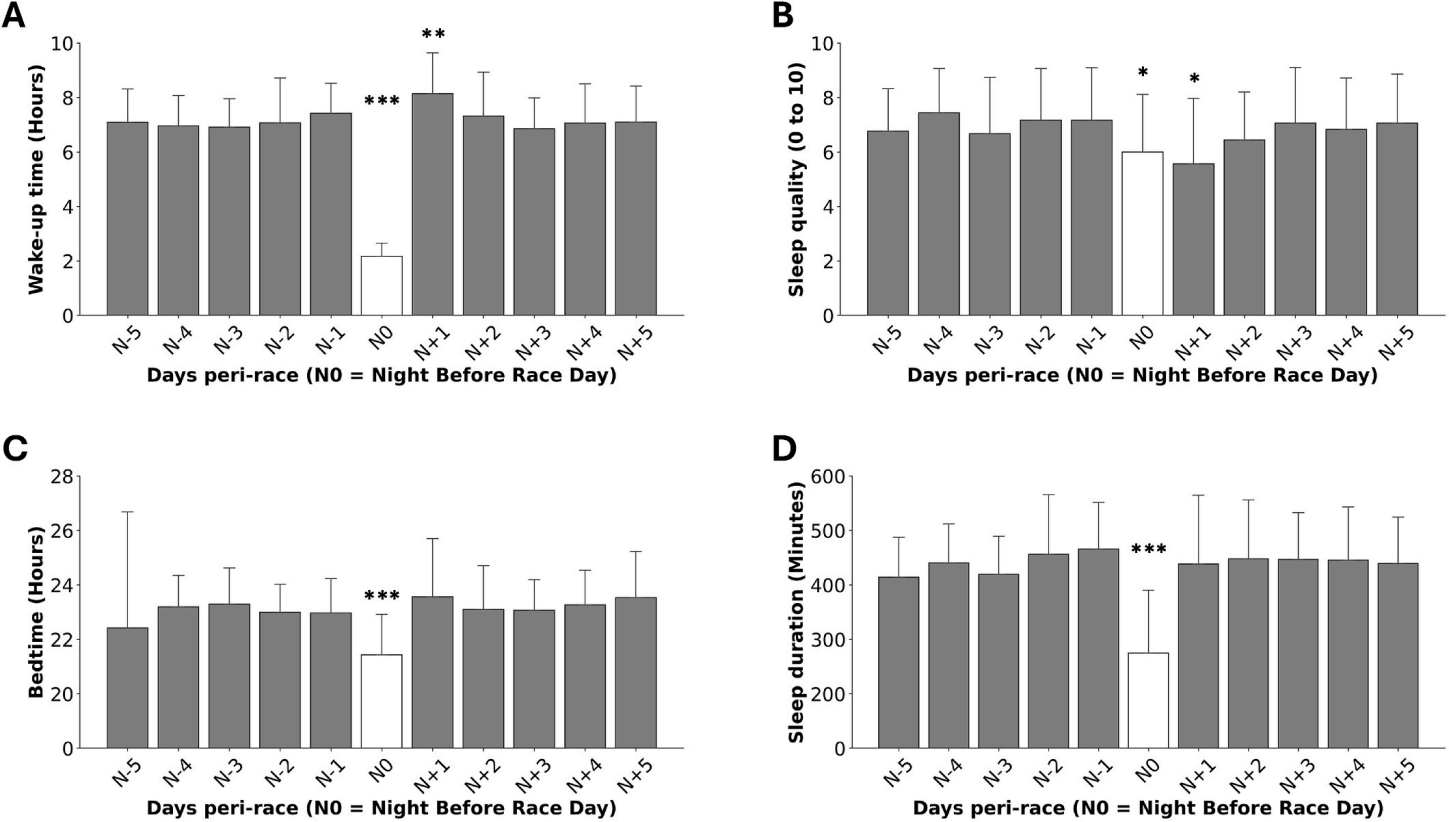
**Sleep changes reported by participants before and after the ultra-trail race.** (A) Wake-up time; (B) Sleep quality; (C) Bedtime; (D) Sleep duration as reported by participants. Sleep diary data are presented as mean ± SD over the 5 d before and 5 d after the race. *p < 0.05; **p < 0.01; ***p < 0.001. Note: Bedtime values exceeding 24 h represent times after midnight (e.g., 25 h = 1:00 a.m.). This avoids discontinuity in the visualisation of bedtime spanning midnight.

Participants reported subjective sleep alterations assessed by sleep diary, including a later wake-up time the morning after the race (N+1, p < 0.01; Fig. 1A) and a poorer sleep quality (N+1, p < 0.05; Fig. 1B) compared to the baseline. There was no change in the time spent in bed (Fig. 1B), but a trend toward increased sleep duration was observed nights before the race (N-1, p = 0.052; Fig. 1D). No other significant results were found for observed variables, excepted on N0 (the night preceding the race). However, these changes were attributed to race-related time constraints (race start at 3 a.m.).

**Fig. 2:**
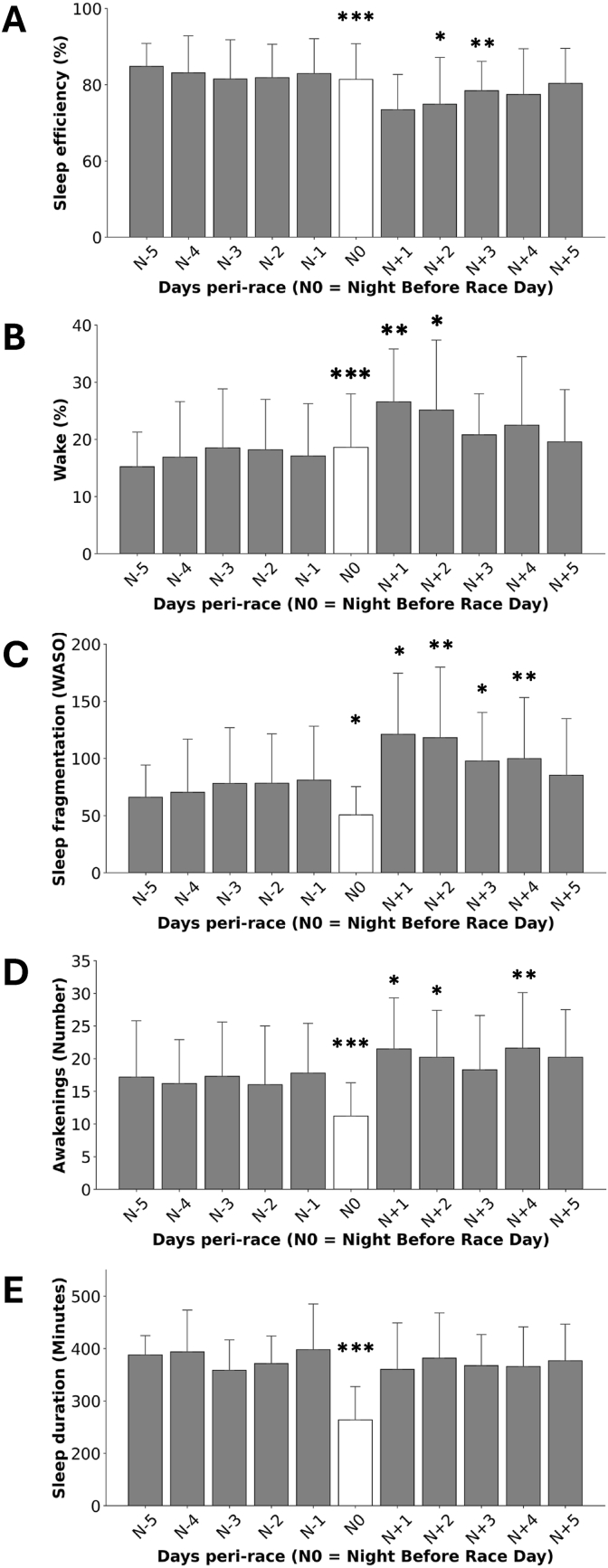
**Sleep changes before and after the ultra-trail running race based on actimetry recordings.** (A) Sleep efficiency (%); (B) Wake percentage after sleep onset (%); (C) Sleep fragmentation, wake after sleep onset (WASO); (D) Number of awakenings; (E) Sleep duration (min). Sleep actimetry data are presented as mean ± SD over the 5 d before and 5 d after the race. *p < 0.05; **p < 0.01; ***p < 0.001.

Actimetry data from at least one night were missing or could not be analysed for five participants. In 13 athletes with complete objective data, sleep efficiency was significantly reduced on the first, second and fourth postrace nights (p < 0.05; Fig. 2A). The sleep continuity was disrupted, with longer wake time on the first and second postrace nights (wake time, p < 0.05; Fig. 2B), accompanied by a significantly increased WASO from the first to fourth night (p < 0.05; Fig. 2C), and a higher number of awakenings (p < 0.05; Fig. 2D). These changes were not accompanied by any change in total sleep duration (p = NS; Fig. 2E).

## 4 Discussion

The current field study sought to assess sleep changes following an ultra-trail running race using subjective measures, such as a sleep diary and objective measures such as actigraphy. We observed the following: 1) the first night after the race was primarily marked by sleep complaints including, a poorer perceived sleep quality and a delayed morning wake-up time; and 2) objectively, sleep became less efficient, particularly on the first night, and remained fragmented with numerous awakenings lasting up to four nights after the race. These measurements revealed profound and lasting changes in sleep after the race, which were poorly reflected in the athletes’ subjective reports, and mainly expressed as a decrease in sleep quality with only minor changes in sleep duration. The origins of these changes, likely multifactorial, remain to be determined.

### 4.1 Potential mechanism underlying sleep disruptions after ultra-trail running

Our results are consistent with existing literature, which report degraded sleep quality following intense and long physical exercise [14,27,50]. In a pioneering laboratory study using polysomnography, Shapiro et al. (1981) had shown that a 92-km ultramarathon increased total sleep time and SW sleep stage duration, a decrease of paradoxical (REM) sleep duration and a more fragmented sleep the following first nights [20]. Along the same lines, laboratory studies showed that physical exercise [14,27,50] as well as the conditions under which they were performed can alter sleep and the homeostatic process involved in sleep regulation [51]. It is well known that sleep deprivation increases homeostatic sleep pressure promoting sleep [52] such as SW and total sleep durations as in this race which begins at 3 a.m. [51,53]. Exposure to hypoxia [54], heat and/or cold [55], in this race reaching an altitude of up to 3000 m additionally challenge sleep regulation and may also contribute to the extension of postrace sleep.

These elements support the hypothesis that SW sleep functions as a key recovery process after ultra-endurance events [56], notably in a race lasting an average of 13.8 ± 5.8 h and involving 5000 m of ascent. However, in our results, the total sleep time with the exception of a later wake-up time following the race was not extended despite the need of recovery. A possible explanation is that for these athletes, social constraints such as work and family obligations from the second day (Monday) onward prevented them from obtaining longer sleep durations. Consequently, an accumulation of sleep debt potentially detrimental to recovery could occur, followed by compensatory sleep increases when possible (e.g., during weekends).

### 4.2 Sleep disruptions after ultra-trail running and recovery

We also observed objective sleep disruptions, mainly reflected by awakenings and the time spent wake after this ultra-trail running race until the fourth night, while most studies indicated a return to normal within 72 h [20,57]. This difference may be explained by the specificity of the event (distance, elevation, altitude) and these observations suggest that these alterations are likely to be more pronounced after intense physical exertion. For example, a study of athletes participating in an ultra-triathlon revealed a significant increase in wakefulness and a reduction in REM sleep compared to days without exercise or after moderate exertion [58].

However, other phenomena need to be considered. Muscle micro-injuries induced by extreme exertion are often correlated with elevated markers of muscle damage such as creatine kinase [27] and an increase in systemic inflammation marked by elevated levels of pro-inflammatory cytokines (IL-6, TNFα) [59]. These mechanisms could be particularly relevant after an event as demanding as the one studied here (64 km with a total of elevation gain and loss). Muscle micro-injuries induced by extreme exertion, evidenced by elevated muscle damage markers (creatine kinase), are correlated with increased sleep fragmentation [27] and systemic inflammation, marked by elevated pro-inflammatory cytokines (IL-6, TNFα), which could also play a major role in sleep changes by promoting awakenings [[60], [61], [62]]. These biological changes may directly affect sleep or indirectly influence it through heightened nociceptive reactivity during sleep causing its disruption [[63], [64], [65]]. These studies show that nociceptive reactivity persists throughout all stages of sleep, with an increased likelihood of awakenings or micro-awakenings, which can impair sleep continuity, as observed in our study. Nociceptive stimuli cause more awakenings (approximately 30% of stimuli) than nonpainful stimuli [66]. Even in the absence of awakening, these stimuli can induce autonomic reactions such as increased heart rhythm, which could contribute to sleep fragmentation and poorer recovery [67].

We hypothesized that two opposing processes are at play: on the one hand, the need for sleep, especially SW sleep driven by energy expenditure [11,20,[51], [52], [53]] and prior sleep deprivation [22,23] and likely other mechanisms [24,25]; and on the other hand, disruptions in sleep continuity caused by pain-related processes [[63], [64], [65]] resulting from biological stress [59,61,62] and alterations in muscles and tendons [27]. Finally, exposure to hypoxia [68] and thermoregulation [69] could also contribute to the extension of postrace sleep.

### 4.3 Implications of sleep changes for daytime activities

These changes are likely to have multiple consequences for daytime activities and the recovery of athletes. It is well known that disrupted sleep lead to impaired vigilance [70]. Indeed, controlled sleep fragmentation protocols have further shown that even preserved total sleep duration can induce vigilance deficits [71]. The observed fragmentation and reduced sleep efficiency may at least partially explain the reported decrease in vigilance for up to one to two weeks postrace in real-life conditions [72], increasing the risk of errors or accidents [57]. In line with this, in ultra-endurance contexts, irregular and insufficient sleep has been associated with impaired vigilance, slower reaction times, altered decision-making, and an increased risk of injury [73]. Accordingly, although sleep deprivation during ultra-trail races may be strategically tolerated during the event, persistent post-race sleep disruptions as those observed in the present study—may compromise safety in the days following competition. Similarly, while the degraded postrace bodily sensations of the musculoskeletal system (such as muscle soreness) and autonomic disturbances (such as vasomotor disorders) are attributable to the physical exercise itself [27,74,75], sleep disruptions related to postrace sleep continuity may also contribute. Indeed, sleep disruptions is known to increase pain sensibility [76] and have been documented to alter autonomic cardiovascular regulation in both the short and long terms [77,78]. Importantly, as muscular recovery is crucial during the postrace period, sleep disruptions may impair postexercise protein synthesis: only one night of sleep deprivation led to an 18% decrease in muscle protein synthesis and was associated with a 21% increase in cortisol levels and a 24% decrease in testosterone [79]. Therefore, facilitating postrace sleep seems essential for optimal recovery. To optimise postexertion recovery, it is essential to create an optimal sleep environment by minimising disruptive stimuli (noise <35 dB, total darkness, temperature between 17 and 28°C) to reduce nocturnal disruptions [80]. Monitoring recovery using actimeters would allow field recovery strategies to be tailored to each individual.

Finally, increasing sleep time before the event may help reduce the effects of sleep deprivation and physical exertion, particularly when chronic sleep deprivation is present. Before the event, consistent with our night before the race (a near significant trend in our study, p = 0.052), Lastella et al. (2015) found that cyclists spontaneously increased their sleep time before major competitions [81,82]; a strategy supported by laboratory findings that highlighted sleep extension on nights before a competition improved attention and reduced sleep pressure [8,83]. Combined with naps, this approach may help reduce sleep pressure and maintain vigilance during exertion [84] and promote the sensation of effort and endurance performance [9]. Adopting these sleep strategies could remain dependent on years of practice [85], particularly for experienced trail and ultra-trail runners [[84], [85], [86]]. These strategies are becoming more widespread [84,86], driven by science communication efforts and trained coaches [87]. However, this strategy does not appear sufficient to fully compensate for postrace sleep debt. Despite no change in total sleep duration after the race, the later wake-up time at first postnight may reflect an insufficient compensatory attempt to restore optimal recovery, as also observed in soccer players [88].

### 4.4 Strengths, limits and perspectives

Several limitations should be noted: the small sample size limits the generalisation and the absence of polysomnography prevents precise assessment of sleep stages, restricting our ability to analyse sleep architecture changes. Sex differences [89,90] and individual chronotypes [91] may influence sleep responses to ultra-trail events and only three participants were female in the present study. We did not assess chronotype, which may limit the generalizability of our findings. This field study entails substantial constraints that account for these limitations but also offers notable strengths. The combination of subjective (sleep diaries, questionnaires) and objective (actimetry) data provides a comprehensive view of sleep alterations. Our study highlights postexertion sleep disruptions, the management of which could help improve athletes’ recovery. Future field studies could help better understand the mechanisms involved by combining polysomnography with inflammatory and biological marker analyses to identify the respective effects of physical exertion, sleep restriction, environmental conditions, and psychological stress on sleep architecture [51,92,93].

## 5 Conclusions

This study revealed that ultra-trail running induces prolonged objective sleep disruptions in athletes, characterised by increased nocturnal wake time and awakenings, along with reduced sleep efficiency, in contrast, sleep duration and sleep complaints showed only minor changes. These alterations persisted for up to five nights after the event. The findings underscore the impact of postrace sleep disruptions and raise questions about the interaction between social and occupational life and recovery in endurance athletes. The underlying mechanisms warrant further investigation to develop strategies that promote optimal recovery in this population.

## Data statement

The data that support the findings of this study are available from the corresponding author upon reasonable request.

## Funding information

This work was supported by a Regional Research Grant from the Réunion Region (Grant No. 324704) and by the European Regional Development Fund (FEDER).

## CRediT authorship contribution statement

**Fanny Fachan:** Conceptualization, Writing – original draft, Writing – review & editing. **Franck Vilcourt:** Conceptualization, Formal analysis, Investigation, Methodology, Validation, Writing – review & editing. **Bruno Lemarchand:** Conceptualization, Formal analysis, Writing – review & editing. **Florian Chouchou:** Conceptualization, Formal analysis, Investigation, Methodology, Project administration, Supervision, Validation, Visualization, Writing – original draft, Writing – review & editing.

## Declaration of competing interest

The authors do not have any conflicts of interest to disclose.
